# RETRACTED ARTICLE: Subsequent anti-myeloma therapy after idecabtagene vicleucel treatment in patients with relapsed/refractory multiple myeloma: A single center analysis

**DOI:** 10.1038/s41408-022-00662-0

**Published:** 2022-04-19

**Authors:** Ricardo D. Parrondo, Keren Sam, Ahsan Rasheed, Victoria Alegria, Taimur Sher, Vivek Roy, Asher Chanan-Khan, Sikander Ailawadhi

**Affiliations:** 1 Division of Hematology-Oncology, Mayo Clinic, Jacksonville, FL USA; 2 Department of Cancer Biology, Mayo Clinic, Jacksonville, FL USA; 3 Hematology-Oncology, St. Vincent’s Riverside, Jacksonville, FL USA

**Keywords:** Cancer immunotherapy, Targeted therapies

The authors have retracted this Correspondence article because, after publication, they became aware that it inadvertently contains some data from a manuscript in preparation that will report the outcome of this clinical trial in full. In addition, as this article contains potentially identifying information, the content is no longer available online in order to protect patient confidentiality. All authors agree to this retraction.

